# Human Fibroblast Switches to Anaerobic Metabolic Pathway in Response to Serum Starvation: A Mimic of Warburg Effect

**Published:** 2014

**Authors:** Monireh Golpour, Haleh Akhavan Niaki, Hamid Reza Khorasani, Arian Hajian, Roya Mehrasa, Amrollah Mostafazadeh

**Affiliations:** 1 *Cellular and Molecular Biology Research Center, Babol University of Medical Sciences, Babol, Iran.*; 2 *Student Research Committee, Babol University of Medical Sciences, Babol, Iran.*; 3 *Blood Transfusion Research Center, High Institute For Research And Education In Transfusion Medicine, Tehran, Iran.*

**Keywords:** Fibroblast, serum starvation, lactate, warburg effect

## Abstract

Fibroblasts could be considered as connective tissue cells that are morphologically heterogeneous with diverse functions depending on their location and activity. These cells play critical role in health and disease such as cancer and wound by Production of collagen, fibronectin, cytokines and growth factors. Absence of insulin and other growth factors in serum deprivation condition and similarity of this condition to the environment of tumor cells and ulcer made us to investigate anaerobic glycolysis in these cells. To this end, we cultured fibroblasts isolated from fresh human newborn foreskin in serum free medium for 16, 24, 48 and 72 hrs and measured glucose consumption, lactate secretion and intracellular LDH in these cells. The results showed despite the lack of insulin, the 16hr serum starved fibroblasts consumed glucose similar to non-starved fibroblasts control. Moreover, in this condition these cells secreted higher levels of lactate and exhibited higher levels of intracellular LDH in comparison to non-starved fibroblasts control. Thus it could be concluded that in serum starvation condition, the newborn human dermal fibroblasts may change the metabolic strategy to Warburg effect. This finding opens a new perspective to further understanding the basic mechanisms involved in communication between tumor cells and fibroblasts.

Fibroblast is the main component of connective tissue and is found almost everywhere in the body. Fibroblasts display diverse morphology and function according to their environments (1). This cell plays an important role in health and disease by production of collagen, fibronectin, cytokines and growth factors (2-6). A bilateral relation between normal epithelial cells and connective tissue components protects the integrity of the natural physiological systems (7-8) In pathological conditions such as cancer or ulcer, the elements of connective tissue specially fibroblast cause epithelial cell proliferation and differentiation by paracrine signaling pathways (9-10), moreover, the bilateral relation between cancer cells and fibroblasts surrounding the tumor plays a role in tumor survival (11-12) Many studies have shown low-nutrient environments are commonly found in the region of tumor and wound and have an important role in gene expressions, angiogenesis, metabolism, and etc., in this condition, cancer associated fibroblasts help cancer cells to growth and metastasis (13-16). Cancer cells also induce anaerobic glycolysis in their associated fibroblasts called reverse Warburg effect (11, 17-18). These mechanisms can cause tumor recurrence, metastasis, and drug resistance in all types of human breast cancer (19-20).

Our previous findings showed that serum starved fibroblast exhibits significantly a higher proliferation rate more than the non-starved control after re-feeding (21). The absence of insulin and other growth factors in this condition and the similarity to the environmental condition of fibroblasts surrounding tumor cells and ulcer, made us to investigate anaerobic glycolysis in these cells.

## Materials and Methods

**Human newborn dermal fibroblast isolation and culture**

Isolated fibroblast based on Pandamoz et al.'s method (22), from six human newborn foreskins at 1-3 months of age that underwent routine circumcision during January 2012 to June 2012 in Amirkola Children Hospital, Babol/ Iran.

**Fibroblast serum starvation shock**

Fibroblast at passage 3-5 were seeded in five 25 ml flasks with Dulbecco's Modified Eagle Medium (DMEM) (PAA cat: E15-883, Austria) + 10% FBS (PAA cat: A 15-15, Austria) + 1% PenStrep® (100μg/ ml) (PAA cat: P11-010, Austria) at a density of 1 x 10^5^ cells per flask and incubated at 37˚C in a humidified condition, 5% CO2, 95% atmospheric air. After reaching fibroblast to 70-80% confluence, removing the supernatant and washing three times with D-phosphate buffered saline (PBS) (PAA cat: H15-002, Austria) then the completed medium was replaced in 4 flasks by DMEM only and the cells were allowed to grow for 16, 24, 48 and 72 h. The sixth flask cells were incubated with DMEM+ 10% FBS as non- starved control. At the end of every period of time, we took the microscopic images of fibroblast and the supernatant of serum starved cells were collected and stored at -20˚ C for further analysis.

**Insulin measurement in cell culture supernatant**

To investigate the metabolism of fibroblasts in serum free condition, first, we examined the presence of insulin in the starved cell culture supernatant. The insulin level of starved fibroblast cell culture supernatant (n=3) was determined by chemiluminescence commercial kit (DiaSorin, Saluggia, Italy).

**Measurement of glucose consumption level**

The 16hr starved fibroblast glucose consumption analysis by GLUCOSE GOD/PAP kit was based on colorimetric method using glucose standard. The glucose levels of starved fibroblast culture supernatant (n=6) as well as DMEM-only were determined by GLUCOSE GOD/ PAP kit(Pars Azmoon, Iran). Then, the DMEM-only glucose level was subtracted from the level of each sample and the result was considered as glucose consumption for each time point.

**Measurement of intracellular lactate dehydrog-enase**

The levels of lactate dehydrogenase (LDH) in cell lysate were determined in an automatic analyzer (COBAS MIRA). Three cell lysates of different indicated times of starved fibroblast were pooled and prepared using the standard protocol (23). The cell lysate protein extraction solution was supplemented with Protease Inhibitor Cocktail (Roche, USA).

**Measurement of lactate in cell culture supernatant**

The level of Lactate in starved and non starved fibroblast cell culture supernatant was determined in an automatic analyzer (COBAS MIRA). The level of starved and non-starved fibroblast culture supernatant (n=3) lactate as well as DMEM+10%FBS was determined by automatic analyzer. Then, the DMEM+10%FBS level was subtracted from the level of non-starved cell culture supernatant and the result was considered as lactate production by non-starved fibroblast and compared with the lactate level of starved fibroblast supernatant.

**Statistical analysis**

Data were represented as mean±SD. Statistical analysis was performed using two-sample, unpaired two-tailed Student’s t-test using Microsoft office Excel 2007 software. Graphs were drawn using Microsoft office Excel 2007 software. A value of p<0.05 was considered statistically significant.

## Results

**Cytoplasmic spreading and cell shape changes in starved fibroblast**

**Fig. 1:**
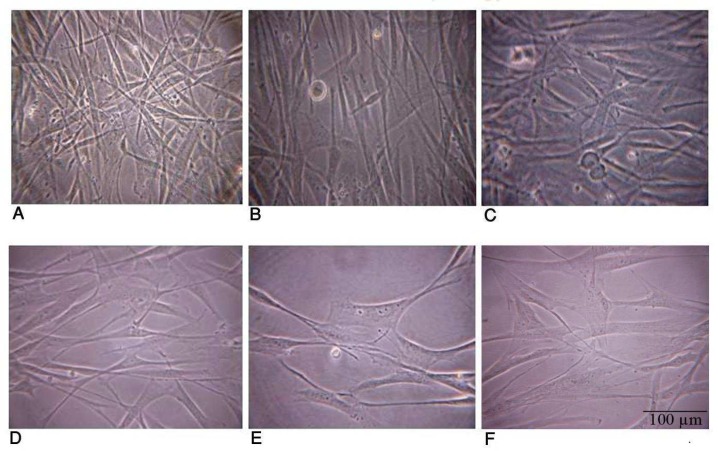
Cytoplasmic spreading and cell shape changes in starved fibroblast. Phase contrast microscopic image (40X) showed non - starved fibroblasts have spindle shape and distinct border (A). 6 and 16 hr starved fibroblast also have similar shape to non-starved fibroblast (B, C). After 24 hr starvation, although the cells are shiny and alive, the more serum starvation lasts, the more cytoplasmic surface will be wide and their borders will be indistinct (D, E, and F

As it can be obviously seen in figure 1 after 24 hr, the starved fibroblast progressively spread its cytoplasm and consequently changed its shape probably to adapt to metabolically unfavorable condition induced by serum starvation. In figure 1, nonstarved and the 6, 16 hr starved fibroblast have spindle shape and distinct border. After 24 hr starvation, the more serum starvation lasts, the more cytoplasmic surface will be wide and their borders will be indistinct but the cells are shiny and alive even up to 72 hrs serum starvation.

**Starved fibroblast has high glucose consumption despite the absence of insulin**

**Fig. 2:**
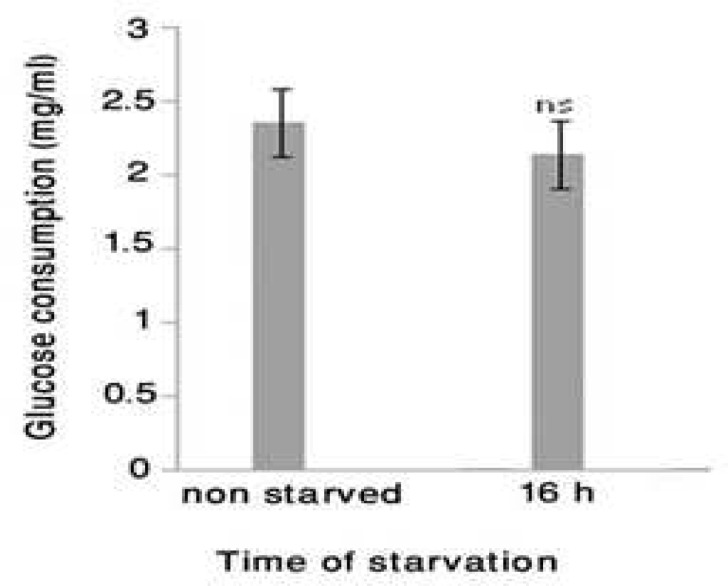
Glucose consumption level of 16 hrs starved fibroblast. The glucose level of 16-hr starved fibroblast culture supernatant (n=6) as well as DMEM-only were determined by GLUCOSE GOD/PAP kit based on colorimetric method. Then, the glucose value of DMEM-only was subtracted from the level of each sample and the result was considered as glucose consumption. The results showed the level of glucose consumption. At this time, there was not a significant difference with non starved fibroblast glucose consumption. The results showed mean ± SD, n=6,*=P<0.05, ns= non significant, and the error bars indicate standard deviation

We were not able to detect insulin at a significant level in starved fibroblast culture supernatants even by routine clinical laboratory test based on very sensitive chemiluminescence technique. Neverthel-ess, after the assessment of glucose consumption by 16 hrs starved fibroblast, the results showed (Fig 2) the level of glucose consumption. At this time point it did not show any significant difference with non-starved fibroblast glucose consumption.

**level of intracellular LDH Increased during serum starvation**

**Fig. 3:**
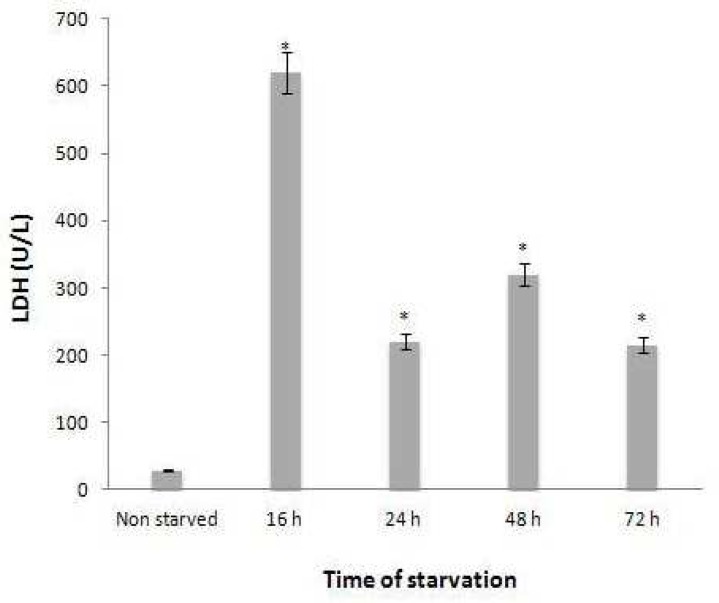
Level of LDH in cell lysate. Three cell lysate samples collected at each time after fibroblast starvation were pooled and the level of LDH was determined using routine COBAS automation method. The data showed the lactate dehydrogenase concentration increased during serum starvation and in the 16-hr starved fibroblast, it was twenty-fold more than the non-starved control (*=P<0.05).

The level of lactate dehydrogenase (LDH) in starved and non-starved fibroblast cell lysate was determined using routine clinical biochemistry laboratory method. The data showed (Fig 3) lactate

dehydrogenase concentration increased during serum starvation and in the 16-hr starved fibroblast, it was twenty-fold more than the non-starved control.

**Serum starved fibroblasts increased lactate production**

**Fig. 4:**
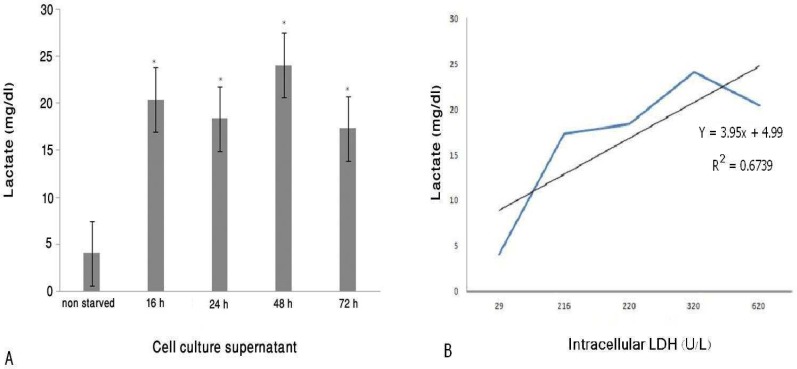
The level of Lactate in fibroblast cell culture supernatant (mean ± SD, n=3). A) The level of lactate in starved and non-starved fibroblast culture supernatant (n=3) as well as DMEM+10% FBS were determined by automatic analyzer (COBAS MIRA). The Produced lactate level by non-starved fibroblast was compared with the produced lactate level by starved fibroblast at each time point. The results showed that the significant level of lactate was detected in starved fibroblast culture supernatant on each point. The results showed as,*=p<0.05, and the error bars indicate standard deviation. B) Direct correlation between intracellular LDH and secreted lactate in starved fibroblasts

The measurement of lactate level in starved fibroblast cell culture supernatant showed (Fig 4-A) the significant level of lactate detected in starved fibroblast culture supernatants at each time point. As shown in figure 4-B, there is a direct correlation between intracellular LDH and secreted lactate in starved fibroblast with R^2^=0.67.

## Discussion

Increased lactate secretion by fibroblast cultured in serum free DMEM is the most important finding in this study. There was a significant difference in lactate secretion activity between the 16, 24, 48 and 72 hrs starved fibroblast and non-starved control cell. The amount of secreted lactate in 48 hours post serum starvation has been increased to about 6 times more than the fibroblasts cultured in complete medium. Our other interesting observation was, despite the lack of insulin in the culture medium, fibroblast cultured in serum free DMEM for 16 hours consumed the glucose, similar to the non-starved control fibroblast. It means that the secreted lactate could originate from the increase in glycolysis pathway activation. We also showed that intracellular LDH levels were increased by serum starvation and reached to their maximum levels after 16 hr. In 16-hr starved fibroblast lysate, this increase was about twenty times more than the non-starved control. As expected, there was also a direct correlation between intracellular LDH levels and lactate concentrations in starved fibroblasts culture supernatants. We detected the highest level of lactate in culture supernatant of the 48-hr starved fibroblast whereas, the 16-hr starved fibroblast exhibited the highest levels of intracellular LDH. We suppose that the 16-hr starved fibroblast consumed the lactate produced by itself to provide energy needed for higher cell metabolism that probably existed at in this time point of the serum starvation protocol. This conclusion is supported by our recent observation that 16hrs fibroblast produces highest levels of protein in comparision to other fibroblast cultured in same condition but for 6, 24, 48, 72 hrs (21).

Our other observation showed that after 24 hr, the starved fibroblast progressively spread its cytoplasm and consequently changed its shape to epithelial-like cell probably to adapt to the unfavorable metabolical condition induced by serum starvation. As fibroblasts are diverse in morphology and function (1), these cells also can be considered metabolical as cells with appropriate plasticity to adapt to the different nutritional microenvironments. Most probably fibroblasts benefit fron this morphological diversity to adapt to a metabolical condition induced by serum starvation, through increasing the cell surface contact with culture plate to obtain many more nutrients and growth factors to warrant the survival of the cell in unfavorable condition such as serum starvation.

Our other observation showed newborn serum starved fibroblasts may do "Warburg effect". The cancer cells even with enough oxygen supply select the anaerobic glycolysis pathway for supplying energy that called Warburge effect (24-25). In our study, it seems that the starved cells selected anaerobic glycolysis pathway for energy production despite of the presence of enough oxygen.

Previously, it has been shown that the cancer-associated fibroblasts also produce lactate through Warburg effect that leads to growth and tumor cell survival even in the absence of angiogenesis (11-12). Also, a similar study showed quiescent cancer associated fibroblasts (CAFs) exhibit high anaerobic metabolic activity despite stopping replication (26). 

Many studies have shown the lactate produced in the "Warburg effect" can create an acidic microenvironment regulating some gene expressions (27-31), for example, at early stage of wound healing process, lactate increment can induce collagen and VEGF synthesis by fibroblasts which in turn can promote wound healing, (32).

Moreover, there are previous reports that demonstrated newborn human fibroblast behave like tumor cells in vitro (33-35). Our findings may also confirm this subject. Thus, it could be concluded that in serum starvation condition, the newborn human dermal fibroblasts may change their metabolic strategy to Warburg effect. This finding opens a new perspective to further understanding of the basic mechanisms involved in communication between tumor cells and fibroblasts.
